# Attention-based message passing and dynamic graph convolution for spatiotemporal data imputation

**DOI:** 10.1038/s41598-023-34077-z

**Published:** 2023-04-27

**Authors:** Yifan Wang, Fanliang Bu, Xiaojun Lv, Zhiwen Hou, Lingbin Bu, Fanxu Meng, Zhongqing Wang

**Affiliations:** 1 School of Information Network Security, People’s Public Security University of China, Beijing, 100038 China; 2 Institute of Computing Technology, China Academy of Railway Sciences Corporation Limited, Beijing, 100081 China

**Keywords:** Computer science, Scientific data, Statistics

## Abstract

Although numerous spatiotemporal approaches have been presented to address the problem of missing spatiotemporal data, there are still limitations in concurrently capturing the underlying spatiotemporal dependence of spatiotemporal graph data. Furthermore, most imputation methods miss the hidden dynamic connection associations that exist between graph nodes over time. To address the aforementioned spatiotemporal data imputation challenge, we present an attention-based message passing and dynamic graph convolution network (ADGCN). Specifically, this paper uses attention mechanisms to unify temporal and spatial continuity and aggregate node neighbor information in multiple directions. Furthermore, a dynamic graph convolution module is designed to capture constantly changing spatial correlations in sensors utilizing a new dynamic graph generation method with gating to transmit node information. Extensive imputation tests in the air quality and traffic flow domains were carried out on four real missing data sets. Experiments show that the ADGCN outperforms the state-of-the-art baseline.

## Introduction

Many application areas have faced significant challenges as a result of missing data. For example, in weather and traffic, missing data from the data gathering process due to sensor failure or network failure will have a significant impact on the output of downstream operations. Many real-world settings have incomplete data that must be processed. As a result, several studies on spatiotemporal data filling methods, such as statistical ways^1^ to fill, machine learning filling methods, and deep learning filling methods, have arisen. In this study, an effective spatiotemporal approach using deep learning to reconstruct the missing parts of the signal produced better filling results. The rise of graph neural networks has also facilitated the development of approaches for imputation that utilize spatiotemporal correlation. Andrea Cini et al. proposed GRIN^2^, which uses a bivariate graph RNN to rebuild missing data in different channels of a multivariate time series by learning the spatiotemporal representation through message passing. However, in the case of this imputation approach, node attributes fluctuate over time rather than being constant at one level. For example, in an air quality collection network, the air quality in a given area depends not only on the prior air quality in that area, but also on the air quality index of the nearby area throughout the previous time. This is because it also takes time for air to travel from one area to another. The same is true for traffic flows in the transport sector. As a result, capturing the spatial correlation of nodes through time and harmonising temporal and spatial continuity has emerged as a significant challenge in the field of data complementation. Zonghan Wu et al. proposed TraverseNet^3^ to address this issue, which employs an attention technique to pick key neighborhood information and deal with dynamic spatiotemporal relationships. There are also several graph neural networks that parse graphs using attention processes^4^. To handle the traffic flow prediction problem, for example, the Spatio-Temporal Graph Convolutional Network with Attention (ASTGCN)^5^ model is utilized. A new direction is the building and update of all surrounding nodes to fill in missing spatiotemporal data using an attention method.

However, in order to capture underlying spatial relationships, numerous similar studies have mapped out deeper graph topologies by creating various adjacency matrices. These methods do not fully exploit the dynamic character of spatiotemporal data, because spatial information changes with time, making it difficult to capture hidden spatial connections. Therefore, the construction of dynamic adjacency matrices becomes a good way to capture spatiotemporal features. For example, Aoyu Liu and Yaying Zhang (2022) presented STIDGCN^6^, an interactive dynamic graph convolution structure formed by merging adaptive and learnable adjacency matrices.

In summary, this paper proposes ADGCN, a dynamic graph attention reconstruction model implemented with a unified spatiotemporal message passing layer. This model first uses the attention mechanism to construct a special messaging layer that assigns each node at each moment a weight value corresponding to its influence. The weights are used to connect each nearby node to the central node, aggregating each neighboring node's state information to update the graph. To reconstruct the spatiotemporal deficiency graph, the model additionally employs a dynamic, adaptive adjacency matrix to learn time-varying correlations between nodes, an adjacency matrix containing static distances, and graph convolution with gating.

The main contributions of this paper are as follows:

- In this paper, an attention-based message passing and dynamic graph convolution network (ADGCN) is proposed. The Message Passing Layer in this architecture can traverse messages from any node at any time, unifying the continuity of time and space.
- This paper presents the dynamic graph convolution module in the model ADGCN. Dynamic graphs are generated by fusing a dynamic adaptive adjacency matrix with a static distance adjacency matrix. It also uses gating to pass information and learn spatial correlations between nodes over time for imputation.
- Extensive experiments were conducted on four real data sets in two domains. The experimental results show that the model ADGCN proposed in this paper has the most advanced performance compared to the baseline model.

The rest of the paper is structured as follows: Section “Related work” describes related work in the area of data imputation. The proposed spatiotemporal reconstruction model ADGCN is described in detail in Section “Methods”. The experimental procedures and results are described in Section “Experiment”. Finally, conclusions are drawn in summary in Section “Conclusion”.

## Related work

### Filling of spatiotemporal data

A large literature exists on the filling of missing values in spatiotemporal data. This includes simple statistical methods that look for overall characteristics of the data or similarities between time series to fill in missing values. Examples include simple mean fill (MEAN), chain multiple interpolation (Azu et al. MICE)^7^, etc. There are also methods that consider spatial information for filling, such as matrix factorisation (Cichocki and Phan, MF)^8^, etc.

However, in the field of machine learning, the filling of spatiotemporal data can be viewed as a prediction task. It fills in the missing values by predicting the missing position data. Among them, there are several variants of supervised algorithms for prediction imputation, including the K-nearest neighbor algorithm (KNN) (Troyanskaya et al.^9^; Beretta and Santaniello^10^), the vector autoregressive algorithm (VAR), and others. These algorithms do not necessarily work very well compared to simple statistics. None of them fully account for the spatiotemporal properties of spatiotemporal data, and predictions must be utilized globally to fill in the gaps.

All current advanced spatiotemporal data filling methods employ deep learning algorithms. Among them, recurrent neural networks (RNN) and their variants^11,12^ based on recursive neural networks are able to achieve better results in the complementation task. Cao et al.^13^ proposed the BRITS model, an RNN-based bidirectional GRU with gating structure for multivariate time series imputation that takes into account the correlation between different channels to perform spatial imputation. Furthermore, Miao et al.^14^ proposed rGAIN, a generative adversarial model based on a bidirectional recurrent neural network, in which the encoder-decoder imputes missing input using a bidirectional RNN. There are also methods that use attention mechanisms to focus on time-series relationships for completeness. Wenjie Du^15^ and others used self-attention based on diagonal masking to explicitly capture temporal dependencies and feature correlations between time steps.

### Graph neural networks

Graph neural networks (GNNs) have always had a unique advantage in processing spatiotemporal data^16–19^. The graph structure in GNN^20^ can efficiently gather spatiotemporal information and update the graph by employing the graph's adjacency matrix and the graph's convolution operation to fuse the information of neighboring nodes. The standard graph neural network only considers static spatial information. For example, GraphSAGE (Hamilton, Ying, and Leskovec 2017)^21^ defines fixed neighbors and aggregates each node's neighborhood and own attributes. Wu et al.^22^ proposed a graph-wave network that uses extended one-dimensional convolution to learn static graph structures from input with spatial dependencies. When both spatiotemporal features are present, spatiotemporal graph neural networks (STGNN) can handle data efficiently. Weiguo Zhu^23^ proposed CorrSTN, a graphical model for traffic flow prediction based on spatiotemporal correlation information. The attention-based mechanism of STGNN can then model time and space separately, extracting time–space dependencies. Chuanpan Zheng et al.^24^ proposed that GMAN captures spatial correlation with temporal correlation using multiple spatiotemporal attention modules. Wei Shao et al.^25^ proposed a new dynamic multi-graph fusion model that fuses multi-graph information by modeling intra-graph and inter-graph node interactions via spatial and graph attention techniques. In a research work on data filling using graph structures, Indro Spinelli et al.^26^ proposed a graph convolutional network fused with generative adversarial networks to fill in missing data. Emanuele Rossi^27^ and Jiaxuan You et al.^28^ used graph representation learning to deal with missing data.

## Methods

**Figure 1:**
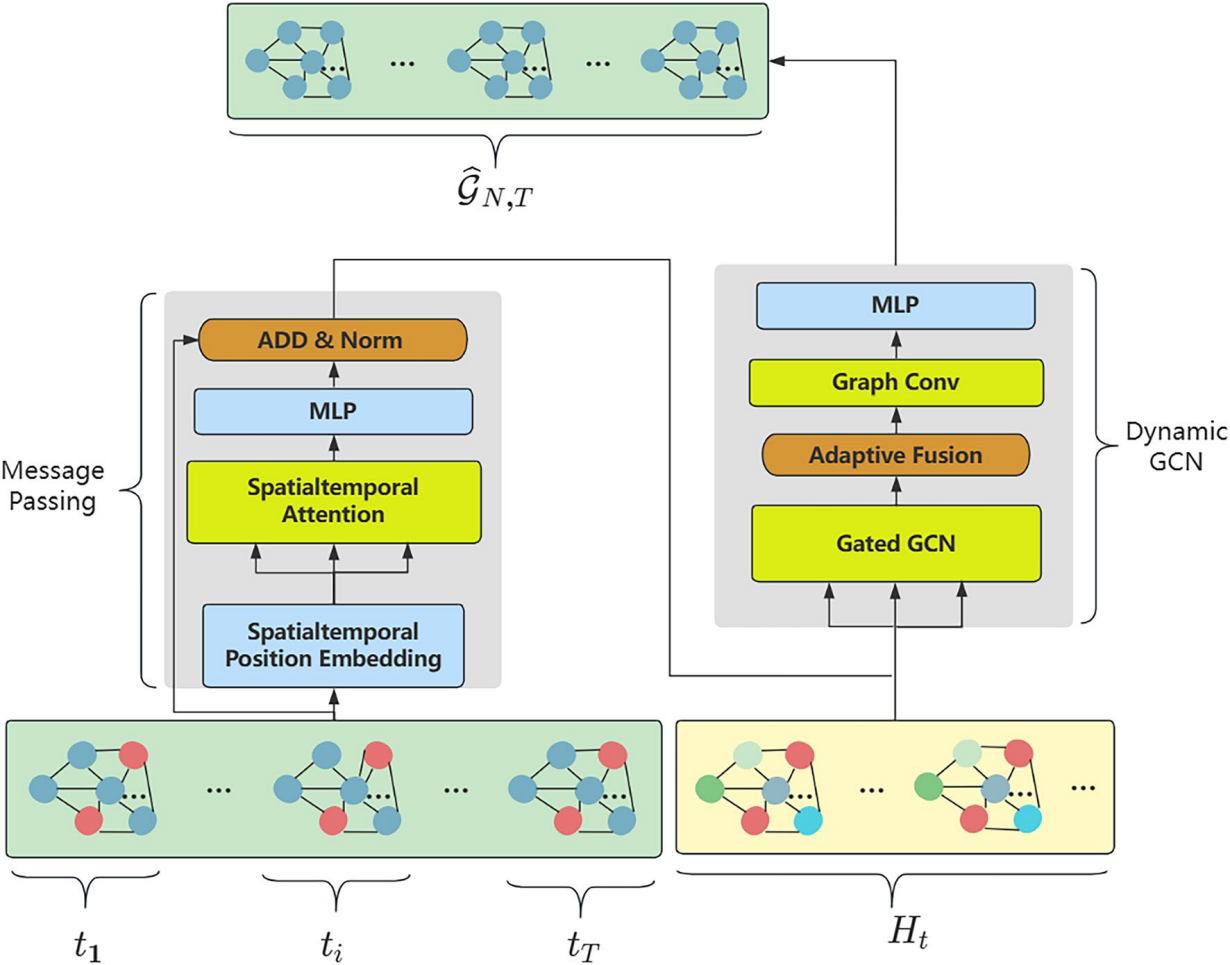
ADGCN Model architecture.

The architecture of the ADGCN model proposed in this paper is shown in Fig. 1 and consists of two main parts: (1) A unified spatiotemporal messaging layer constructed using attention mechanisms; (2) Dynamic graph convolution method using dynamic adjacency matrix in conjunction with static adjacency matrix, and gating to pass information. The detailed steps for each of these two sections are described separately below.

**Figure 2:**
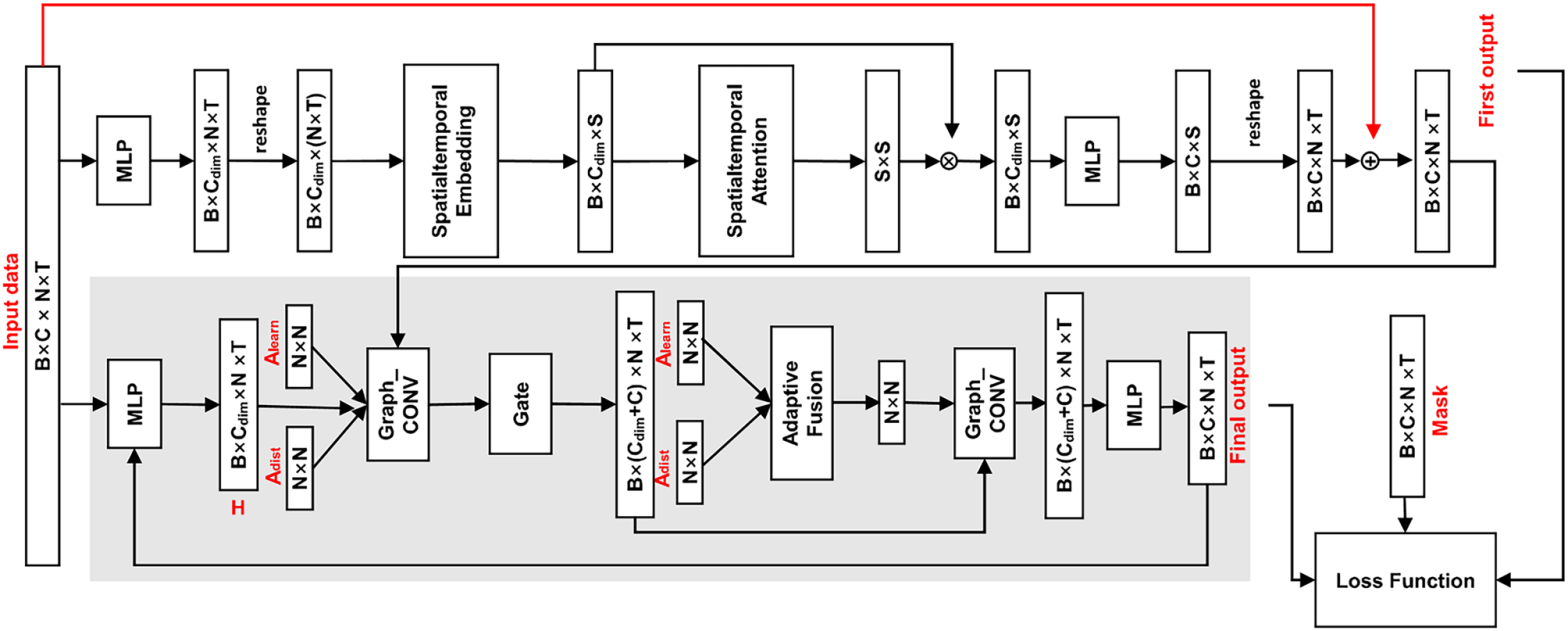
ADGCN model architecture.

The architecture of the full model implementation is shown in Fig. 2, together with the vector dimensions of the model inputs and outputs. Where $B$ represents the batch size, $C$ represents the number of channels 1, ${C}_{dim}$ represents the hidden layer dimension, $N$ represents the sensor spatial dimension, $T$ represents the temporal dimension, and $S$ is the spatiotemporal dimension that fuses the temporal and spatial dimensions, $S=N\times T$. The unified spatiotemporal messaging layer and the dynamic graph convolution filling method with gating are the two key components that constitute the model.

First the raw data is input to the unified spatiotemporal messaging layer, of which the most significant module is the spatiotemporal attention module in Fig. 2. We firstly map the data using spatiotemporal embedding to extract its spatiotemporal features in order to unify the continuity of time and space. The spatial dimension and the temporal dimension are then combined to create the spatiotemporal component. After that, the spatiotemporal attention matrix is derived from the spatiotemporal attention, and the significance of each node is updated by merging the spatiotemporal component inputs. Ultimately, $MLP$ is able to determine the original dimension. To reduce the loss of information in the raw data, we use the residuals to add the raw data to the output, as shown by the red arrows in Fig. 2.

The second network module is our proposed gated dynamic graph convolution layer, of which the most prominent module is the graph convolution layer in Fig. 2. The input is a concatenation of the output sequence of the previous network and the original sequence embedded by $MLP$ with the spatiotemporal representation $H$, which is the original data mapped by $MLP$. To aggregate the node information, this representation is convolved with each of our two suggested adjacency matrices for gated graphs. Then, in order to update the node information, we perform a dynamic graph convolution operation with the output of the adaptive fusion of the dynamic adjacency matrix with the static adjacency matrix. Finally, the final reconstructed sequence is output using $MLP$ to derive the complemented data. When processing the next sequence of graphs, we re-enter the output data through the $MLP$ embedding as the spatiotemporal representation $H$ at that point into the dynamic gated graph convolution layer.

### Data pre-processing and concepts

It begins with a description of how the data is processed and the definition of some concepts. Given a collection of multivariate time series with T time steps and N-dimensional sensors, consider the sensors in the data as nodes and construct a graph $G=(\mathrm{V},\mathrm{E})$. Where $V$ represents the set of $N$ sensors at the nodes in the graph, ${v}_{i}\in V$ is a node. $\varepsilon ={\{{e}_{ij}\}}_{i,j=1}^{N}$ denotes the relationship between nodes. If nodes ${v}_{i}$ and ${v}_{j}$ are adjacent, then ${e}_{ij}=1$; otherwise, ${e}_{ij}=0$. Define the raw data of the i-th sensor as ${X}_{i}=({x}_{i1},{x}_{i2},{x}_{i3},\dots ,{x}_{iT})$, where ${x}_{it}$ denotes the data in time interval t for the i-th sensor. $T$ indicates the length of the data for each sensor. To represent the missing variables in $X$, introduce the missing mask vector $M={\left\{{m}_{i}\right\}}_{i=1}^{N},M\in {R}^{T\times N}$.

1 $${M}_{t}^{d}=\left\{\begin{array}{c}1\, if\, {X}_{t} \,is\, observed\\ \,0 \,if\, {X}_{t}\, is\, missing\end{array}\right.$$

where ${m}_{it}$ is zero if the data ${x}_{it}$ is missing, and one if ${x}_{it}$ is observed.

**Figure 3:**
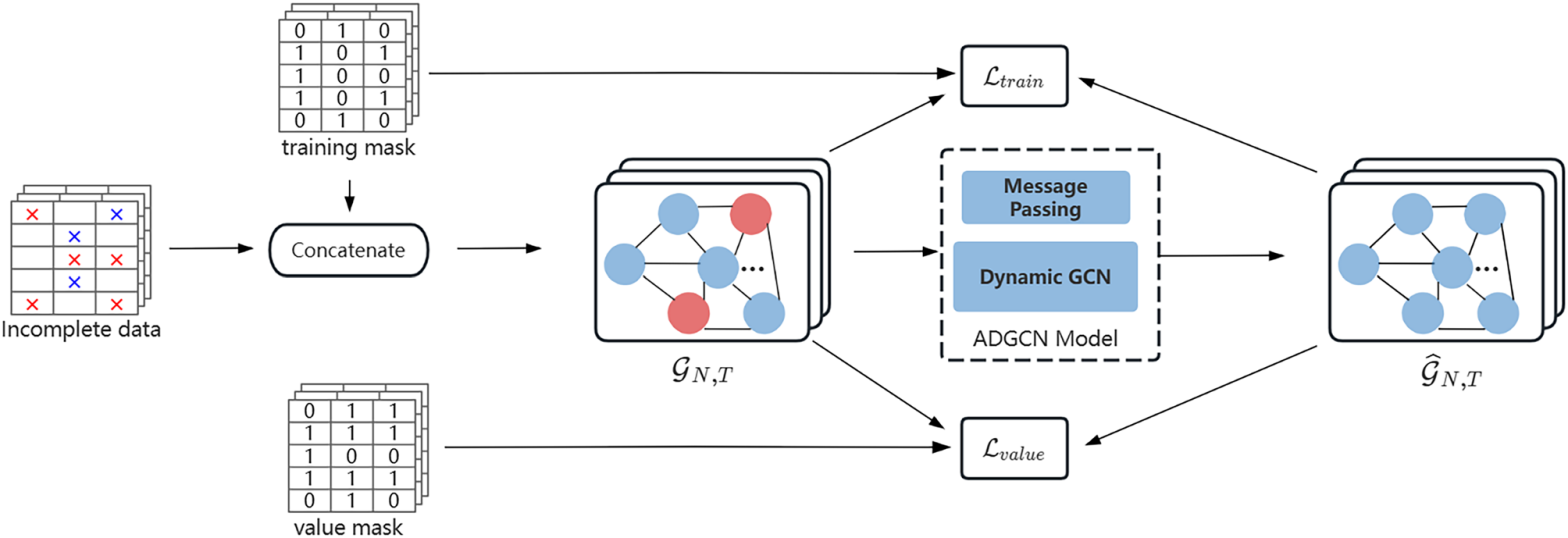
Description of the training method for reconstructing missing map sequences.

Figure 3 shows the construction of the graph and the training method for filling the data. To begin, the red forks in the incomplete data reveal the real missing data, whereas the blue forks represent missing values generated at random for training. In this paper, we construct a graph sequence ${\mathcal{G}}_{N,T}$ with sensors as nodes and reconstruct the data using the proposed model. We define the reconstruction error ${\mathcal{L}}_{train}, {\mathcal{L}}_{val}$ uniformly as:

2 $$\mathcal{L}\left({\widehat{X}}_{N,T},{\widetilde{X}}_{N,T},{M}_{N,T}\right)=\sum_{t=1}^{T}\frac{{\sum }_{i=1}^{N}{m}_{it}\cdot \ell({\widehat{x}}_{it},{\widetilde{x}}_{it})}{{\sum }_{i=1}^{N}{m}_{it}}$$

where $\ell(\cdot ,\cdot )$ represents the element-level error function (using absolute or squared error), ${\widetilde{X}}_{N,T}$ represents the incomplete data node matrix, ${\widehat{X}}_{N,T}$ is the reconstructed complete data matrix, and ${M}_{N,T}$ represents the mask matrix. Formally, the objective of multivariate time series filling is to find the estimate ${\widehat{X}}_{N,T}$ that minimizes the reconstruction error. During training, we should randomly simulate the presence of deficits in order to better train the reconstruction task.

### Unified spatiotemporal message passing layer

In dealing with the missing problem, the current advanced filling method is to use graph neural networks for filling. Traditional graph convolution or message passing layers, on the other hand, only transfer node information in space, commonly modeling time and space separately and then fusing the information in time and space using techniques like weighing. This situation, on the other hand, is bound to result in information loss and irregular learning dynamics.

**Figure 4:**
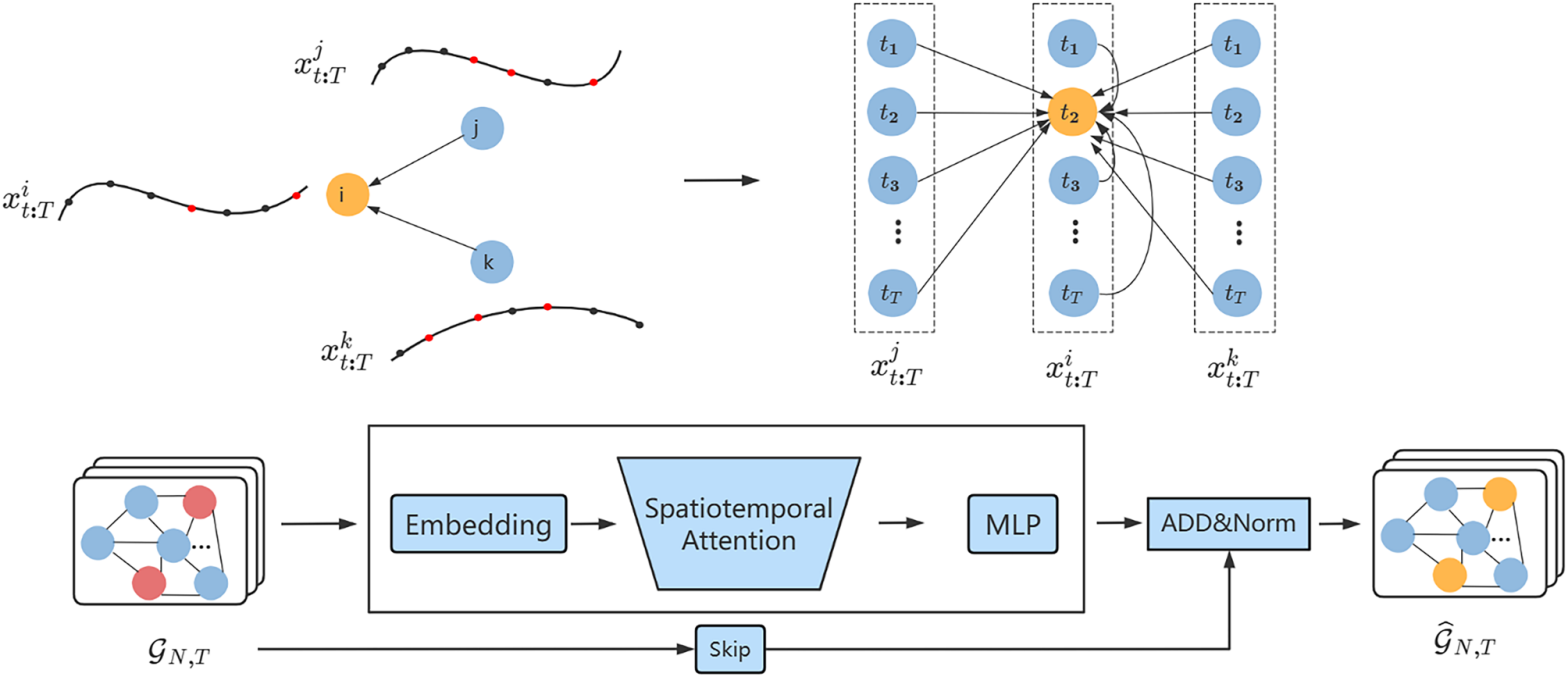
The messaging layer of the unified space–time.

This paper introduces a new message passing layer for spatiotemporal graph neural networks in order to avoid fragmentation of the time–space continuum and to achieve direct transfer of information in time and space. By modeling each sensor at each moment as a node of the graph, an attention mechanism is used to calculate the neighboring nodes to enable message passing. Figure 4 shows an example of inter-neighbor messaging using the moment ${t}_{2}$ of one of the sensors $i$. Long-term spatiotemporal dependencies must be considered, as there are effects between different sensors at different points in time. For example, the ${t}_{2}$ moment of sensor $i$ will be influenced not only by the ${t}_{2}$ moments of the other sensors $(j,k)$, but also by the other moments of $j$ and $k$. For example, traffic jams on one road may generate congestion on another nearby road some time later; rainfall in one area can lead to rainfall in another area some time later. This shows that treating spatial and temporal correlation separately is inappropriate.

To overcome these problems, this paper proposes a message-passing layer that can unify space–time and enable nodes to sense messages from their neighbors over time. An attention mechanism is used to assign corresponding weights to neighboring nodes and focus on the states of more similar neighboring nodes. The spatiotemporal graph is reconstructed by efficiently passing the node's neighborhood information and establishing a past-to-present connection for each neighbor. This paper uses an attention mechanism to aggregate the set of node information ${x}_{t:T}^{i}$ at different moments of the same sensor with the set of node information ${x}_{t:T}^{j}$ of its neighbors to learn to reconstruct the complete data representation. Figure 4 illustrates this learning process.

Where ${x}_{t:T}^{i}$ is the temporal data of the ith sensor T time length, and the set of sensors ${\mathcal{X}}_{t:T}=({x}_{t:T}^{1},{x}_{t:T}^{2},\dots ,{x}_{t:T}^{i},{x}_{t:T}^{j},\dots ,{x}_{t:T}^{N})$ form the graph ${\mathcal{G}}_{N,T}$. The sequence of missing graphs is reconstructed after a message passing layer with spatiotemporal attention as the core.

Initially encoding the data as high-dimensional data and extracting significant spatiotemporal features.

3 $$\widetilde{\mathcal{X}}=MLP(\mathcal{X})$$

Update node i's information for ${x}_{t:T}^{i}$ in $\widetilde{\mathcal{X}}$ from time t = 0 to time t = T:

4 $${\widetilde{x}}_{t:T}^{i}=\sum_{j\in N(u)}\alpha ({x}_{t:T}^{j},{x}_{t:T}^{i})W{x}_{t:T}^{i}$$

where $\alpha (\cdot ,\cdot )$ is the attention function:

5 $$\alpha ({x}_{t:T}^{j},{x}_{t:T}^{i})=\frac{\mathrm{exp}({x}_{t:T}^{j}{w}_{j}||{x}_{t:T}^{i}{w}_{i})}{{\sum }_{i\in N(u)}\mathrm{exp}({x}_{t:T}^{j}{w}_{j}||{x}_{t:T}^{i}{w}_{i})}$$

where W is the model parameter.

6 $${\widehat{X}}_{t:T}^{i}={x}_{t:T}^{i}+MLP({\widetilde{x}}_{t:T}^{i})$$

Eventually, use aggregation as well as residual blocks to reconstruct the incomplete data ${\widehat{\mathcal{X}}}_{t:T}=({\widehat{X}}_{t:T}^{1},{\widehat{X}}_{t:T}^{2},\dots ,{\widehat{X}}_{t:T}^{i},\dots ,{\widehat{X}}_{t:T}^{N})$. The reconstructed graph sequence now goes through the missing mask vector $M$ to fill in the missing position data in order to obtain the complete feature vector ${\widehat{X}}_{t:T}^{`}$.

7 $${\widehat{X}}_{t:T}^{`}={\mathcal{X}}_{t:T}\odot M+(1-M)\odot {\widehat{\mathcal{X}}}_{t:T}$$

### Dynamic graph convolution filling method with gating

**Figure 5:**
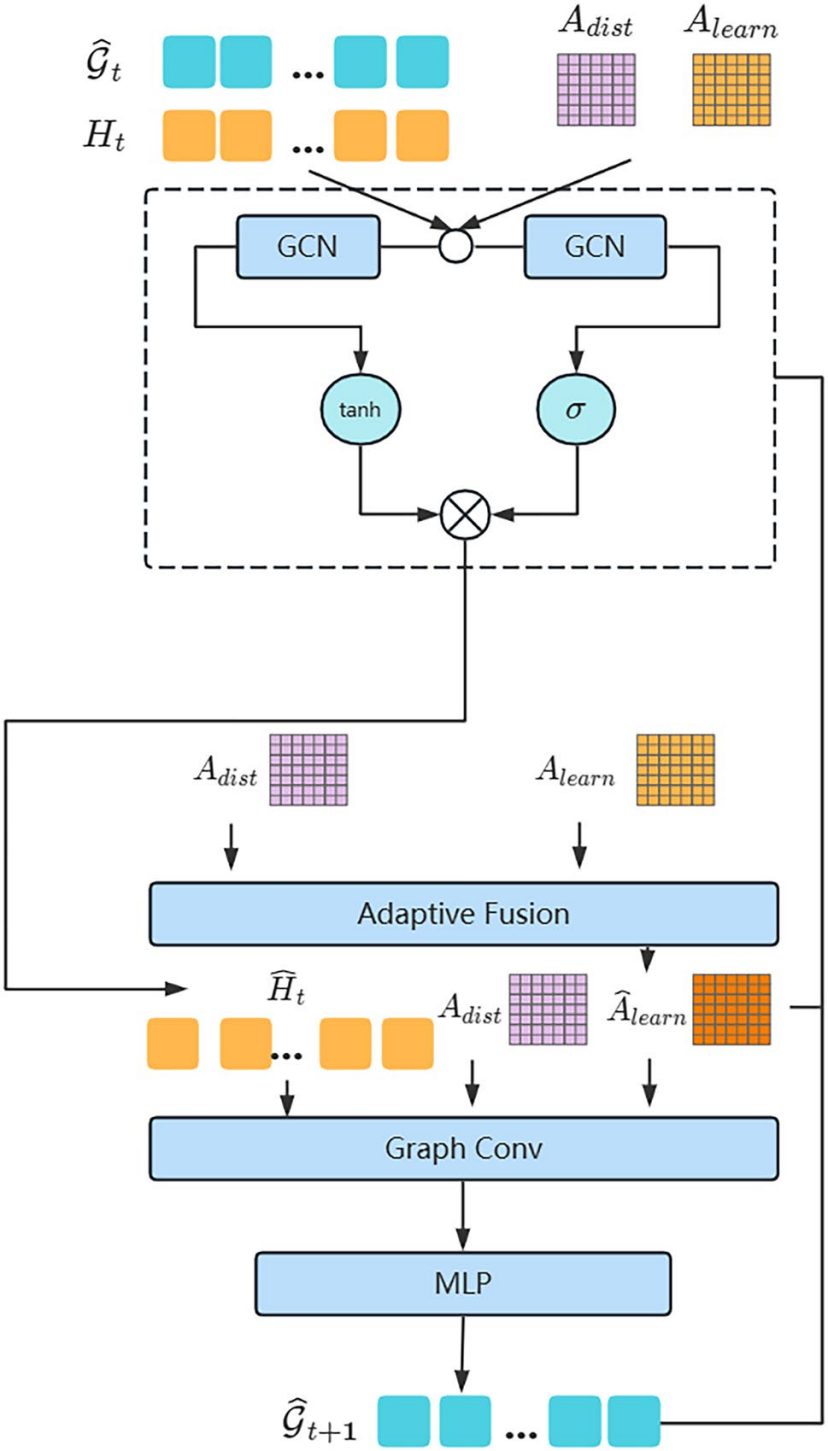
Dynamic graph convolution layer with gating.

Following the message passing layer, the output ${\widehat{X}}_{t:T}^{`}$ will be utilized as the input for the next component to be learned. For graph convolution, this part will use a combination of building dynamic adjacency matrices and using distances as static adjacency matrices. As shown in Fig. 5, the input sequence is mapped to the space–time representation ${H}_{t:T}\in {R}^{N\times l}$. Two adjacency matrices are also defined: one is a static adjacency matrix ${A}_{dist}\in {R}^{N\times N}$, using the geographical distance between sensors; the other is a dynamic adjacency matrix ${A}_{learn}\in {R}^{N\times N}$ that can be learned. Simultaneously, the connected sequence ${\widehat{X}}_{t:T}^{`}$, the mask ${M}_{t:T}$, and the spatiotemporal representation ${H}_{t:T}$ are fed into a graphical convolutional neural network with a modified gating function, which processes the sequence separately in forward and reverse directions, one step at a time, to update and reconstruct the data. For the adjacency matrix, we use the same forward and backward transfer matrices as the adjacency matrix A, denoted ${A}_{f}=A/rowsum(A)$ and ${A}_{b}={A}^{T}/rowsum({A}^{T})$, respectively.

This will then be fed into a gating graph convolutional neural network. This paper incorporates a learning module for time and space by replacing the GRU's fully connected layer with a graph convolution layer that selectively maintains information from the previous time step and updates the current sequence.

8 $${r}_{t}^{i}=\sigma \left(GCN\left([{A}_{dist},{A}_{learn}],\left[{\widehat{x}}_{t}^{i}\left|\left|{m}_{t}^{i}\right|\right|{h}_{t-1}^{i}\right]\right)\right)+{b}_{tr}$$

9 $${u}_{t}^{i}=\sigma \left(GCN\left([{A}_{dist},{A}_{learn}],\left[{\widehat{x}}_{t}^{i}\left|\left|{m}_{t}^{i}\right|\right|{h}_{t-1}^{i}\right]\right)\right)+{b}_{tu}$$

10 $${c}_{t}^{i}=tanh\left(GCN\left([{A}_{dist},{A}_{learn}],\left[{\widehat{x}}_{t}^{i}\left|\left|{m}_{t}^{i}\right|\right|{r}_{t}^{i}*{h}_{t-1}^{i}\right]\right)\right)+{b}_{tc}$$

11 $$h_{t}^{i} = u_{t}^{i} \odot h_{t - 1}^{i} + \left( {1 - u_{t}^{i} } \right) \odot c_{t}^{i}$$

where ${r}_{t}^{i}$ and ${u}_{t}^{i}$ are the reset and update gates respectively, ${c}_{t}^{i}$ is the cell state information, and ${\widehat{x}}_{t}^{i}$ is the output of the previous time step. The symbols $\odot$ and || denote the Hadamard product and concatenation operators respectively. The reset gate ${r}_{t}^{i}$ determines how the new input information is combined with the previous information, and the update gate ${u}_{t}^{i}$ is used to control the extent to which the state information from the previous moment is brought into the current state. The computation of ${h}_{t}^{i}$ is to forget some dimensional information in ${h}_{t-1}^{i}$ passed down and to update it by adding some dimensional information input from the current node. The GCN module in the context is defined as:

12 $$GCN(A,{X}_{in})=\sum_{k=0}^{K}\left({A}_{f}^{k}{X}_{in}{W}_{1}+{A}_{b}^{k}{X}_{in}{W}_{2}\right)$$

where A represents the set of adjacency matrices ${A}_{dist}$ and ${A}_{learn}$, and ${X}_{in}$ represents the input sequence. ${W}_{1}$ and ${W}_{2}$ are the model parameters, and $K$ is the number of layers.

Subsequently, we will use the adaptive fusion structure to fuse the static adjacency matrix ${A}_{dist}$ with the dynamic adjacency matrix ${A}_{learn}$ to make adjustments to the dynamic adjacency matrix ${A}_{learn}$. We define the dynamic adjacency matrix ${A}_{learn}$ as:

13 $${A}_{learn}=SoftMax(Relu({N}_{1}{{N}_{2}}^{T}))$$

where ${N}_{1}\in {R}^{N\times c},{N}_{2}\in {R}^{c\times N}$ are the learnable parameters. ${A}_{learn}$ can learn the implicit relationships between graph nodes and enhance the model's ability to capture spatial heterogeneity. However, there is a limit to how much ${A}_{learn}$ can learn, and as the model is trained, ${A}_{learn}$ will be fixed. While the static adjacency matrix defined by spatial distance contains geospatial information, we fuse the adjacency matrix ${A}_{dist}$ with static spatial distance to guide the generation of a dynamic adjacency matrix to capture deeper spatial features.

Subsequently, we fuse ${A}_{learn}$ with ${A}_{dist}$ using the adaptive fusion structure.

14 $${\widehat{A}}_{learn}=\beta {A}_{learn}+(1-\beta ){A}_{dist}$$

where ${\widehat{A}}_{learn}$ is the updated dynamic adjacency matrix and β is the learnable adaptive parameter factor. After fusion, we get the adjacency matrix ${\widehat{A}}_{learn}$.

We take the hidden state output at each time step through the $MLP$ as one fill, i.e., we use the mask ${M}_{t}$ to guide the missing positions and replace ${\widehat{X}}_{t}^{\left(1\right)}$ with the missing position data in the original data ${X}_{t}$ to form the filled data ${\widehat{X}}_{t}$. The sequence then continues to learn the fill function after the fill.

15 $${\widehat{X}}_{t}^{\left(1\right)}=MLP\left({H}_{t-1}\right)$$

16 $$\hat{X}_{t} = M_{t} \odot X_{t} + \left( {1 - M_{t} } \right) \odot \hat{X}_{t}^{\left( 1 \right)} .$$

To update the hidden state ${H}_{t}$, the updated sequence features ${\widehat{X}}_{t}$ and the set of adjacency matrices $A$ are fed back into the gated graphical convolutional neural network. The feature ${\widehat{X}}_{t}$ will be mapped to a new spatiotemporal representation ${H}_{t}$ by an $MLP$. Then continue processing the input graph ${\mathcal{G}}_{t+1}$ for the next time series. This method combines the forward and backward sequences to produce the final output, which is the whole data after reconstruction.

## Experiment

This section evaluates four typical datasets often used in the field of data imputation utilizing current state-of-the-art baselines as well as the method provided in this paper. The final results show that the method proposed in this paper achieves the most advanced performance of all the baselines.

### Datasets

This paper uses real datasets from two domains: an air quality dataset and a transport domain dataset, both with spatiotemporal characteristics. The datasets contain both the sensor timing data and the sensor geolocation data. The constructs were deficient for better training during the experiment. Details of the dataset are shown in the Table 1 (P for Point Missing, B for Block Missing).

**Table 1:** Details relating to the datasets^3,29^.

Dataset	Node	Time step	Missing%	Constructive missing%
AQI-36	36	36	13.24	10.67
AQI	437	24	25.67	11.33
METR-LA (P)	207	24	8.10	23.0
(B)	–	–	–	8.4
PEMS-BAY(P)	325	24	0.02	25.0
(B)	–	–	–	9.07

### Air quality (AQI) dataset

The Urban Computing Project (Zheng et al.^30,31^) has published several datasets, including the impact of air quality on human life. In particular, the Air Quality Index (AQI) dataset contains hourly measurements of six pollutants from 437 air quality monitoring stations in 43 cities in China over a one-year period (from May 2014 to April 2015). Of these, the missing rate reached 25.67%. Yi et al.^32^ conducted imputation experiments in a simplified version of the AQI, and this dataset is also taken into account in this paper. The simplified version (AQI-36) contains data from 36 air quality monitoring stations and has a 13.24% missing rate. For the sake of experiment comparability, the months of March, June, September, and December in the dataset are used as the test set in this paper, as in the GRIN setting. The time step was chosen to be T = 24 in AQI and for AQI-36 a time step of T = 36 was chosen for the experiment. The geographical coordinates of each monitoring point in this dataset will be utilized to generate the static adjacency matrix in the article. To obtain the adjacency matrix from the geographical distance between nodes, we use a threshold Gaussian kernel (SHuman et al. 2013)^33^. For example, the weight ${E}_{i,j}$ of the edge between the i-th node and the j-th node at a certain threshold $\delta$ is defined as:

17 $${E}_{i,j}=\mathrm{exp}\left(-\frac{{dist\left(i,j\right)}^{2}}{d}\right), dist\left(i,j\right)\le \delta$$

where $dist\left(\cdot ,\cdot \right)$ is the calculated geolocation distance function, $d$ is the width of the Gaussian kernel and $\delta$ is the threshold value. To maintain experimental consistency, we set d to the standard deviation of the geographical distances provided in the dataset and $\delta$ to a distance of 40 km.

### Traffic flow datasets

In the field of data imputation, traffic flow data is also commonly used as a dataset to verify the effectiveness of imputation. This paper therefore uses the PEMS-Bay and METR-LA datasets from Li et al.^29^. METR-LA contains 4 months of sensor readings from 207 sensors on Los Angeles County motorways (Jagadish et al. 2014)^34^ with a sampling rate of 5 min; PEMS-BA Y contains 6 months of data from 325 traffic sensors in the San Francisco Bay Area with a sampling rate of 5 min. Both datasets have a time step of 24, i.e. 2 h of data. Similarly, the Leigh dataset uses the provided sensor geolocation data to construct a static adjacency matrix using a threshold Gaussian kernel. This paper will be consistent with the experimental setup in GRIN, using 70% of the data for training, 10% as the validation set and 20% as the test set. Where we simulate the presence of missing data by referring to the settings in GRIN: (1) Block loss, i.e., at each step of each sensor, we discard 5% of the available data at random; (2) Point loss, where we simply mask 25% of the available data at random.

### Baseline approach

The filling method proposed in this paper is complementary in the spatiotemporal dimension and mainly considers other advanced filling methods for comparison: (1) VAR, a vector autoregressive one-step ahead predictor, set with a batch size of 64 and a learning rate of 0.0005, using SGD to train the model to predict the next value using the last five historical observations; (2) SAITS, which uses self-attention to fill in missing data from a time series. For the setting of the hyperparameters, we use the hyperparameter range from the original paper; (3) BRITS, a model that in data with a bidirectional recursive approach. It uses the network hyperparameters adopted for the AQI-36 dataset, with the hidden state size set to 128 in the AQI and METR-LA datasets compared to 256 in the PEMS-BA dataset; (4) E2GAN^35^, an end-to-end generative model to estimate missing values in multivariate time series, with parameter settings consistent with those in BRITS. (5) rGAIN, the GAIN with a bidirectional recursive encoder and decoder, has parameter settings consistent with those found in BRITS. (6) CSDI^36^, a new time series imputation method, the fraction-based diffusion model, which uses a fraction-based diffusion model based on observed data to fill in missing values. (7) GRIN, which reconstructs missing data in different channels of a multivariate time series by learning the spatiotemporal representation through message passing, with the same parameter settings as in the original paper. As well as some simple statistical filler methods: (8) MEAN, mean fill; (9) MICE algorithm, multiple interpolation of chain equations; (10) KNN, K-nearest neighbor algorithm; (11) MF, Matrix decomposition algorithm.

### Experimental setup

The experiments in this paper were implemented using Pytorch, trained and experimentally validated on an NVIDIA TeslaV100 GPU. For the ADGCN algorithm in this paper, the ADAM optimizer was used and trained using the cosine learning rate scheduler. The initial value of the learning rate was 0.001, and 300 rounds were used for training, with 160 randomly sampled batches of 32 elements per round, set in line with GRIN. The evaluation method uses three metrics, MAE (mean absolute error), MSE (mean square error) and MAPE (mean absolute percentage error), to evaluate the performance of the model:

18 $$MAE\left( {imputation,target,mask} \right) = \frac{{\mathop \sum \nolimits_{n}^{N} \mathop \sum \nolimits_{t}^{T} \left| {\left( {imputation_{t}^{n} - target_{t}^{n} } \right) \odot mask_{t}^{n} } \right|}}{{\mathop \sum \nolimits_{n}^{N} \mathop \sum \nolimits_{t}^{T} |mask_{t}^{n} }}$$

19 $$MSE\left( {imputation,target,mask} \right) = \frac{{\mathop \sum \nolimits_{n}^{N} \mathop \sum \nolimits_{t}^{T} \left( {\left( {imputation_{t}^{n} - target_{t}^{n} } \right) \odot mask_{t}^{n} } \right)^{2} }}{{\mathop \sum \nolimits_{n}^{N} \mathop \sum \nolimits_{t}^{T} |mask_{t}^{n} |}}$$

20 $$MAPE\left( {imputation,target,mask} \right) = \frac{{\mathop \sum \nolimits_{n}^{N} \mathop \sum \nolimits_{t}^{T} \left| {\left( {imputation_{t}^{n} - target_{t}^{n} } \right) \odot mask_{t}^{n} } \right|}}{{\mathop \sum \nolimits_{n}^{N} \mathop \sum \nolimits_{t}^{T} |target \odot mask_{t}^{n} |}} \times 100{\text{\% }}$$

### Hyperparameter experiments

**Figure 6:**
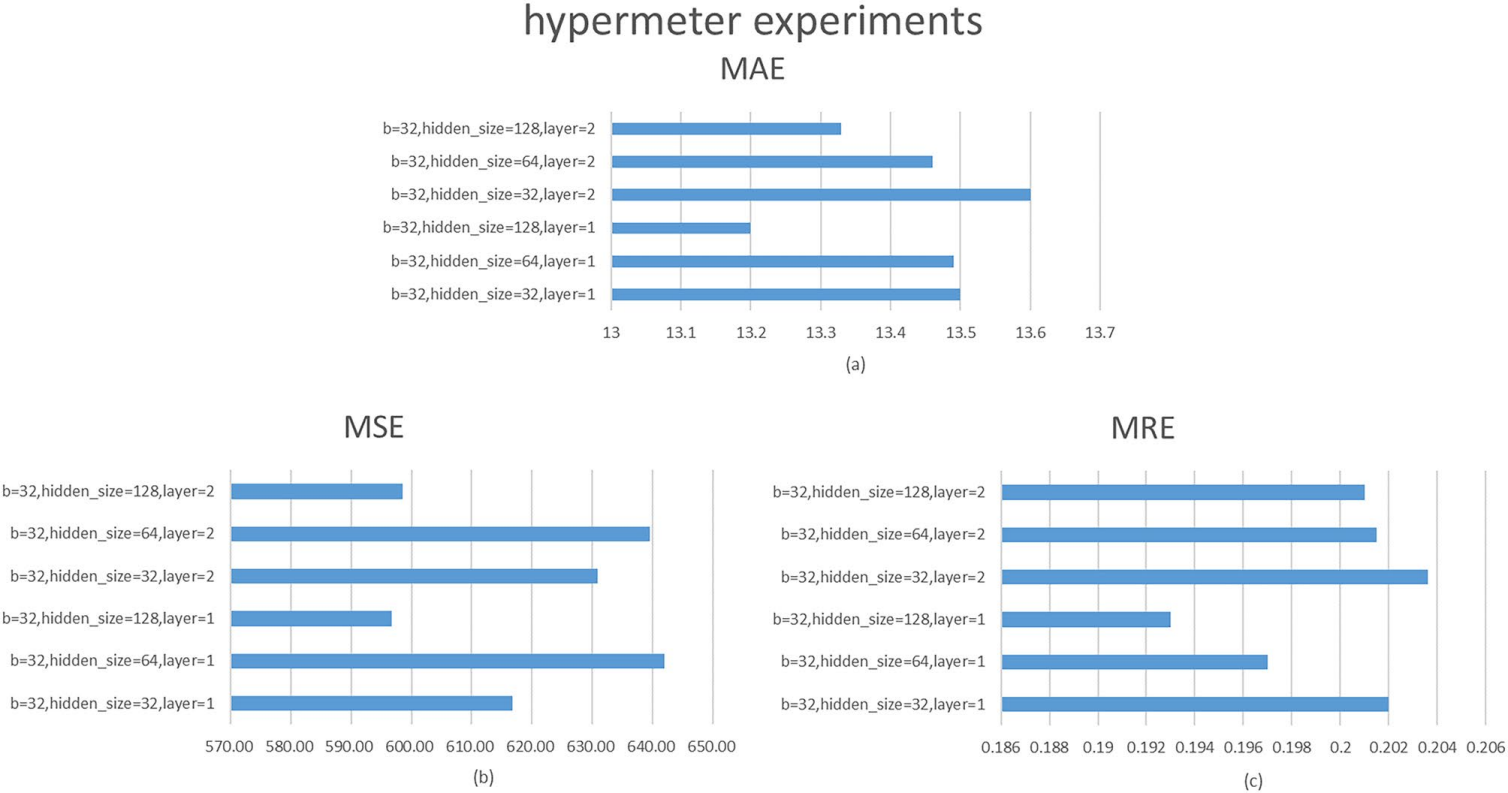
Hyperparameter experimental results.

In this section, we design hyperparameter experiments to find more efficient hyperparameters. The experiments keep the batch size the same as the settings in the other baselines, so we only change the size of the hidden layers in the model with the number of layers of the graph convolution. The number of graph convolution layers is set to 1 and 2, while the hidden layer sizes are set to 32, 64, and 128. Figure 6 shows the results of hyperparametric tests on the AQI dataset for air quality.

Figure 6 shows the results of hyperparametric tests on the AQI dataset for air quality. The model achieves optimal performance when the batch size is 32, the hidden layer size is 128, and the number of graph convolution layers is 1. As a result, this hyperparameter setting will be used for all of our upcoming experiments.

### Experimental results

In this section, the performance of the baseline is compared with the model proposed in this paper on four real data sets from two classical domains. Table 2 shows the experimental results of the model and baseline on the air quality dataset. Table 3 shows the results of the complementary performance on the traffic flow dataset. The results show that the ADGCN outperformed the baseline in all cases.

**Table 2:** Comparison of model performance for an average of 5 experiments filled on the air quality domain dataset.

Model	AQI-36			AQI		
	mae	mse	mape(%)	mae	mse	mape(%)
MEAN^2^	53.48	4578.08	76.77	39.60	3231.04	59.25
KNN^2^	30.21	2892.31	43.36	34.10	3471.14	51.02
MF^2^	30.54	2763.06	43.84	26.74	2021.44	40.01
MICE^2^	30.37	2594.06	43.59	26.98	1930.92	40.37
VAR^2^	15.64	833.46	22.02	22.95	1402.84	33.99
E2GAN	15.78	741.81	22.66	21.52	1240.81	32.21
rGAIN^2^	15.37	641.92	21.63	21.78	1274.93	32.26
BRITS^2^	14.50	662.36	20.41	20.21	1157.89	29.94
SAITS^37^^*^	18.16	843.53	37.16	21.33	1253.23	31.74
CSDI	**9.74**	**383.63**	**11.32**	19.71	1196.59	27.96
MPGRU^2^	16.79	1103.04	23.63	18.76	1194.35	27.79
GRIN^2^	12.08	523.14	17.00	14.73	775.91	21.82
ADGCN	11.93	502.31	17.13	**13.49**	**642.00**	**20.19**

^*^ please note: mse and mape(%) for SAITS are not reported in^37^, but were calculated for this article. The best results are in bold.

**Table 3:** Comparison of model performance for an average of 5 experimental fills on the traffic flow domain dataset.

Model	METR-LA						PEMS-BAY					
	Blocking missing			Point missing			Blocking missing			Point missing		
	mae	mse	mape (%)	mae	mse	Mape (%)	mae	mse	Mape (%)	mae	mse	Mape (%)
MEAN^2^	7.48	139.54	12.96	7.56	142.22	13.10	5.46	87.56	8.75	5.42	86.59	8.67
KNN^2^	7.79	124.61	13.49	7.88	129.29	13.65	4.30	49.90	6.90	4.30	49.80	6.88
MF^2^	5.46	109.61	9.46	5.56	113.46	9.62	3.28	50.14	5.26	3.29	51.39	5.27
MICE^2^	4.22	51.07	7.31	4.42	55.07	7.65	2.94	28.28	4.71	3.09	31.43	4.95
VAR^2^	3.11	28.00	5.38	2.69	21.10	4.66	2.09	16.06	3.35	1.30	6.52	2.07
E2GAN	3.00	23.49	5.21	2.98	22.80	7.99	1.97	12.20	3.16	1.77	9.73	2.83
rGAIN^2^	2.90	21.67	5.02	2.83	20.03	4.91	2.18	13.96	3.50	1.88	10.37	3.01
BRITS^2^	2.34	17.00	4.05	2.34	16.46	4.05	1.70	10.50	2.72	1.47	7.94	2.36
SAITS^37^^*^	2.30	16.88	4.00	2.26	16.32	3.94	1.56	14.02	2.50	1.40	7.88	2.30
CSDI	2.23	15.92	3.64	2.20	14.32	3.42	1.50	13.77	2.50	1.22	7.75	1.82
MPGRU^2^	2.57	25.15	4.44	2.44	22.17	4.22	1.59	14.19	2.56	1.11	7.59	1.77
GRIN^2^	2.03	13.26	3.52	1.91	10.41	3.30	1.14	6.60	1.83	0.67	1.55	1.08
ADGCN	**2.02**	**13.22**	**3.51**	**1.89**	**10.31**	**3.27**	**1.07**	**5.23**	**1.73**	**0.66**	**1.52**	**1.07**

^*^ please note: mse and mape(%) for SAITS are not reported in^37^, but were calculated for this article. The best results are in bold.

The experimental results show that ADGCN achieves the best performance in complementing spatiotemporal data in different scenarios. Statistical approaches are often utilized less efficiently to fill in the data than deep learning methods, as deep learning methods can seek for correlations between data as well as extract temporal and spatial aspects from the data. rGAIN learns the distribution of real data using generative adversarial networks, temporal reminder matrices, and classifiers to estimate missing values that converge to the true data distribution for filling. BRITS is based on recurrent neural networks that directly learn missing values in bidirectional recursive dynamical systems. SAITS uses a self-attentive mechanism to capture temporal dependencies and feature correlations to impute the data. CSDI, on the other hand, used a diffusion model to fill in the data.

Where CSDI produces better results on the AQI-36 dataset with only 36 nodes, our model yields better performance when we switch to the larger AQI dataset with 437 nodes. Also, on other large traffic datasets, our model produces better results in comparison. Because of the spatially distinctive nature of the dataset in this paper, CSDI may not achieve optimal results.

However, E2GAN, rGAIN, BRITS, CSDI, and SAITS ignore the spatiotemporal properties of the data and do not use its spatial properties to fill in, considering only the temporal nature of the data. Furthermore, MPGRU is a GNN-based one-step prediction, similar to DCRNN, on which GRIN is an enhancement, intending to rebuild missing data in distinct channels of a multivariate time series by learning the spatiotemporal representation through message passing. This paper proposes ADGCN to address the fact that the time-dependent cycles of the neighbors of message-passing nodes in GRIN may be delayed or dynamic, severing the continuity between time and space. It can unify the temporal and spatial messaging layers in messaging and can dynamically adapt the adjacency matrix of spatial node neighborhood relationships.

In conclusion, the experimental results show that the ADGCN can capture both temporal and spatial information, achieving the best filling performance in spatiotemporal data imputation tests.

### Robustness experiments

The Tables 1 through 3 show how well the model fills in missing data, however this section will raise the rate of missing data for experimental validation in order to confirm the model's robustness.

**Figure 7:**
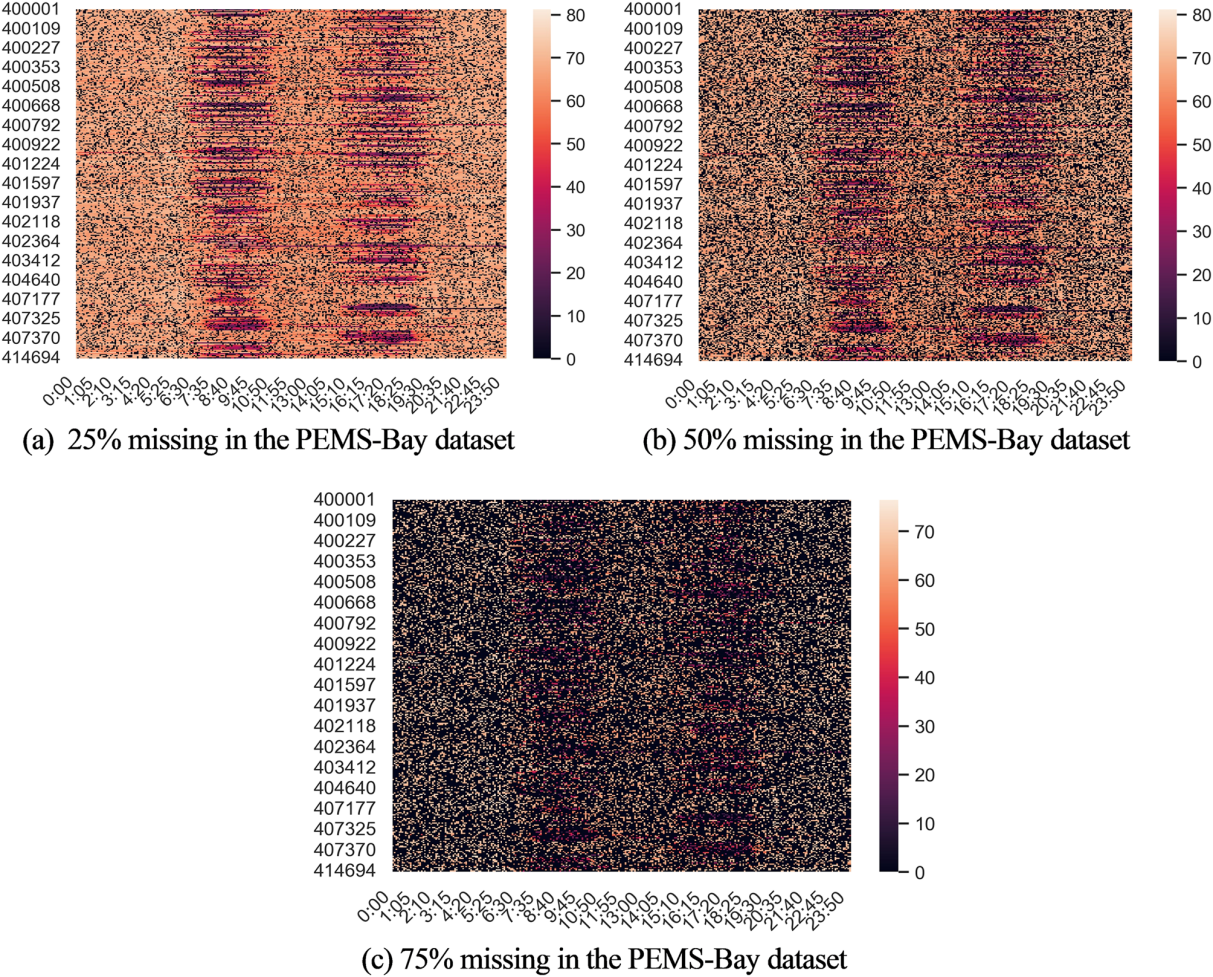
Heat map of the PEMS-Bay dataset at different deletion rates.

The two large traffic speed datasets described above were used as examples for the experiments. As an example, visualize the 25%, 50%, and 75% missing data rate scenarios constructed for every 5 min of data in the San Francisco Bay Area PEMS-Bay traffic dataset for the entire day of May 2, 2015. Figure 7 shows the distribution of the data at different deletion rates in a heat map. While the vertical coordinate denotes the various detectors, the horizontal coordinate denotes the time of day when the data was taken, and the degree of color denotes the various speeds. When the rate of missingness rises, it can be observed that the dataset contains an increasing number of zero items, which are represented by an increasing number of black dots on the heat map. To confirm the stability and robustness of the model, comparison experiments with the baseline were run at various deletion rates. Table 4 shows the results of the robustness experiments.

**Table 4:** Comparison of model MAE results when data absence rate increases.

Model	METR-LA			PEMS-BAY		
	25%	50%	75%	25%	50%	75%
BRITS^37^	2.34	2.52	3.02	1.47	1.55	2.17
SAITS^37^	2.26	2.48	3.74	1.40	1.50	2.96
GRIN^37^	1.91	2.05	2.39	0.67	0.79	1.09
ADGCN	**1.89**	**2.01**	**2.35**	**0.66**	**0.75**	**0.99**

The best results are in bold.

As shown in Table 4, the proposed ADGCN model shows excellent performance in all cases, below the complementary error of other state-of-the-art models. Therefore, the model proposed in this paper has good robustness, and with the increase of the missing rate, the model still shows excellent filling performance.

### Ablation experiments

In this section, the paper uses ablation experiments to verify the effectiveness of the two-part module in the ADGCN. The ADGCN is separated into three components: a message-passing layer without the attention mechanism (DGCN) and a model removing the dynamic graph adjacency matrix (AGCN), as well as the full ADGCN model. In addition, we replace the fully connected layer in GRU with graph convolution in the gated graph dynamic graph convolution module. Therefore, in order to verify the validity of this transformation, we will switch back to the fully connected structure of GRU for experiments to compare with our model. The performance of the four models is compared in Table 5.

**Table 5:** Ablation experiments, MAE values for an average of 5 times, (P) and (B) indicate missing points and blocks missing settings respectively.

Model	AQI-36	AQI	METR-LA(B)	METR-LA(P)	PEMS-BAY(B)	PEMS-BAY(P)
AGCN	13.10	13.84	2.32	2.11	1.21	0.71
DGCN	12.00	13.72	2.08	1.92	1.13	0.67
ADGRU	13.70	14.26	2.52	1.94	1.15	0.68
ADGCN	**11.93**	**13.49**	**2.02**	**1.89**	**1.07**	**0.66**

The best results are in bold.

Since most studies ignore the continuity of time and space, during the model's design process, we established each sensor at each moment as a node of the graph and used the attention mechanism to give each node's neighbor a corresponding weight to update the graph. After the graph was constructed, a dynamic graph convolution module with gating was created to recreate the missing graph sequence via graph convolution of the adjacency matrix, merging static distances with a dynamically adaptive adjacency matrix based on spatiotemporal data.

From the results in Table 5, it is clear that ADGCN cannot obtain the best complementary effect no matter which part is missing. We also replace the fully connected layer of GRU with a graph convolution operation, which can better integrate temporal and spatial information. Experiments show that the use of graph convolution works better.

## Conclusion

This paper proposes a dynamic graph neural network reconstruction model, ADGCN, capable of unifying time and space. This method treats the sensor state at each moment as a node of the graph and updates the graph by giving its weight through an attention mechanism to complete the construction of a unified temporal and spatial messaging layer. And dynamic spatial correlation is represented by a dynamic adaptive module, in which the input spatiotemporal information is used to construct the graph adjacency matrix structure, which is then fused with the defined static distance adjacency matrix. The ADGCN model explores the connections between each node in the network at each point in time in order to capture hidden temporal and spatial correlations and to simulate the spatial dynamics of node correlations. Experiments on four real-world datasets show that combining the temporal and spatial continuums and dynamically constructing spatial node interactions improves data imputation. Additionally, we test various sets of parameter values for experiments during the model's training phase before choosing the best ones, demonstrating that the model ADGCN proposed in this paper outperforms the state-of-the-art baseline. To further verify the robustness of ADGCN, this paper will increase the missing rate on two major traffic datasets to verify the stability of the model. Experimental results show that the imputation performance of the model decreases as the missing rate increases but still yields the best performance compared to the state-of-the-art imputation methods. Therefore the model has good robustness. In addition, this paper uses ablation experiments to verify the validity of each module design in the ADGCN model. The model ADGCN proposed in this paper outperforms the state-of-the-art baseline.

## Supplementary Information

Supplementary Information.

## Data Availability

Data contained within the Supplementary Material is reproduced from https://github.com/Graph-Machine-Learning-Group/grin. The data presented in this study are available in Supplementary Material here.
