# Author Correction: Attention-based message passing and dynamic graph convolution for spatiotemporal data imputation

**DOI:** 10.1038/s41598-023-38722-5

**Published:** 2023-07-21

**Authors:** Yifan Wang, Fanliang Bu, Xiaojun Lv, Zhiwen Hou, Lingbin Bu, Fanxu Meng, Zhongqing Wang

**Affiliations:** 1 School of Information Network Security, People’s Public Security University of China, Beijing, 100038 China; 2 Institute of Computing Technology, China Academy of Railway Sciences Corporation Limited, Beijing, 100081 China

Correction to: *Scientific Reports*, 10.1038/s41598-023-34077-z, published online 27 April 2023

The original Article contained errors in the References and the Data availability sections. The following references were omitted:

[2] Cini, A., Marisca, I. & Alippi, C. Filling the G_ap_s: Multivariate time series imputation by Graph Neural Networks. International Conference on Learning Representations, ICLR (2022)

[37] Marisca, I., Cini, A. & Alippi, C. Learning to Reconstruct Missing Data from Spatiotemporal Graphs with Sparse Observations. Advances in Neural Information Processing Systems, NeurIPS (2022)

As a result, all subsequent references were renumbered.

In addition, Reference [20] contained an error in the author list. The original reference [20] is shown below.

[20] Dao, M. *et al*. Scalable Spatiotemporal Graph Neural Networks. (2022).

It now reads:

[20] Cini, A., Marisca, I., Bianchi, F.M., & Alippi, C. Scalable Spatiotemporal Graph Neural Networks, NeurIPS (2022)

In the Introduction section, the following sentence was missing a reference to [2]:

Andrea Cini *et al*. proposed GRIN, which uses a bivariate graph RNN to rebuild missing data in different channels of a multivariate time series by learning the spatiotemporal representation through message passing.

Now reads:

Andrea Cini *et al*. proposed GRIN [2], which uses a bivariate graph RNN to rebuild missing data in different channels of a multivariate time series by learning the spatiotemporal representation through message passing.

The original Table 1 was missing references to [2] and [33]. The original Table 1 and its accompanying legend are shown below.

**Table 1:** Details relating to the datasets.

Dataset	Node	Time step	Missing%	Constructive missing%
AQI-36	36	36	13.24	10.67
AQI	437	24	25.67	11.33
METR-LA (P)	207	24	8.10	23.0
(B)	–	–	–	8.4
PEMS-BAY(P)	325	24	0.02	25.0
(B)	–	–	–	9.07

The original Table 2 was missing references to [2] and [37]. The original Table 2 and its accompanying legend are shown below.

**Table 2:** Comparison of model performance for an average of 5 experiments filled on the air quality domain dataset.

Model	AQI-36			AQI		
	mae	mse	mape(%)	mae	mse	mape(%)
MEAN	53.48	4578.08	76.77	39.60	3231.04	59.25
KNN	30.21	2892.31	43.36	34.10	3471.14	51.02
MF	30.54	2763.06	43.84	26.74	2021.44	40.01
MICE	30.37	2594.06	43.59	26.98	1930.92	40.37
VAR	15.64	833.46	22.02	22.95	1402.84	33.99
E2GAN	15.78	741.81	22.66	21.52	1240.81	32.21
rGAIN	15.37	641.92	21.63	21.78	1274.93	32.26
BRITS	14.50	662.36	20.41	20.21	1157.89	29.94
SAITS	18.16	843.53	37.16	21.33	1253.23	31.74
CSDI	**9.74**	**383.63**	**11.32**	19.71	1196.59	27.96
MPGRU	16.79	1103.04	23.63	18.76	1194.35	27.79
GRIN	12.08	523.14	17.00	14.73	775.91	21.82
ADGCN	11.93	502.31	17.13	**13.49**	**642.00**	**20.19**

The best results are in bold.

The original Table 3 was missing references to [2] and [37]. The original Table 3 and its accompanying legend are shown below.

**Table 3:** Comparison of model performance for an average of 5 experimental fills on the traffic flow domain dataset.

Model	METR-LA						PEMS-BAY					
	Blocking missing			Point missing			Blocking missing			Point missing		
	mae	mse	mape (%)	mae	mse	Mape (%)	mae	mse	Mape (%)	mae	mse	Mape (%)
MEAN	7.48	139.54	12.96	7.56	142.22	13.10	5.46	87.56	8.75	5.42	86.59	8.67
KNN	7.79	124.61	13.49	7.88	129.29	13.65	4.30	49.90	6.90	4.30	49.80	6.88
MF	5.46	109.61	9.46	5.56	113.46	9.62	3.28	50.14	5.26	3.29	51.39	5.27
MICE	4.22	51.07	7.31	4.42	55.07	7.65	2.94	28.28	4.71	3.09	31.43	4.95
VAR	3.11	28.00	5.38	2.69	21.10	4.66	2.09	16.06	3.35	1.30	6.52	2.07
E2GAN	3.00	23.49	5.21	2.98	22.80	7.99	1.97	12.20	3.16	1.77	9.73	2.83
rGAIN	2.90	21.67	5.02	2.83	20.03	4.91	2.18	13.96	3.50	1.88	10.37	3.01
BRITS	2.34	17.00	4.05	2.34	16.46	4.05	1.70	10.50	2.72	1.47	7.94	2.36
SAITS	2.30	16.88	4.00	2.26	16.32	3.94	1.56	14.02	2.50	1.40	7.88	2.30
CSDI	2.23	15.92	3.64	2.20	14.32	3.42	1.50	13.77	2.50	1.22	7.75	1.82
MPGRU	2.57	25.15	4.44	2.44	22.17	4.22	1.59	14.19	2.56	1.11	7.59	1.77
GRIN	2.03	13.26	3.52	1.91	10.41	3.30	1.14	6.60	1.83	0.67	1.55	1.08
ADGCN	**2.02**	**13.22**	**3.51**	**1.89**	**10.31**	**3.27**	**1.07**	**5.23**	**1.73**	**0.66**	**1.52**	**1.07**

The best results are in bold.

The original Table 4 was missing references to [37]. The original Table 4 and its accompanying legend are shown below.

**Table 4:** Comparison of model MAE results when data absence rate increases.

Model	METR-LA			PEMS-BAY		
	25%	50%	75%	25%	50%	75%
BRITS	2.34	2.52	3.02	1.47	1.55	2.17
SAITS	2.26	2.48	3.74	1.40	1.50	2.96
GRIN	1.91	2.05	2.39	0.67	0.79	1.09
ADGCN	**1.89**	**2.01**	**2.35**	**0.66**	**0.75**	**0.99**

The best results are in bold.

The Data availability section was:

Data is contained within the Supplementary Material. The data presented in this study are available in Supplementary Material here.

It now reads:

Data contained within the Supplementary Material is reproduced from https://github.com/Graph-Machine-Learning-Group/grin. The data presented in this study are available in Supplementary Material here.

The original Article has been corrected.

